# Supplementary material for: Gut microbiota and its influence on the Gut-Brain axis in comparison with chemotherapy patients and cancer-free control data in Breast cancer—A computational perspective
Source: PLoS One. 2025 Jun 3;20(6):e0324742. doi: 10.1371/journal.pone.0324742 (PMC12132997; doi:10.1371/journal.pone.0324742)
Supplement: Attachment — Submitted filename: Response to Reviewer_PONE-D-25-05321_R1.docx [file pone.0324742.s004.docx]

We humbly thank the reviewer for raising key concerns in the article and helping to improve the quality of the manuscript. We have incorporated all the improvements in the revised manuscript, and we strongly believe that the revised version would be more suitable to publish in your journal. The incorporated changes are marked with the Red font. Responses to the reviewer's comments are enumerated below, and appropriate changes have been made in the revised manuscript.

**Reviewer #1:**

**Comment 1:** The study analyzes only 33 samples (19 CFC, 14 DT), which may not provide enough statistical power to draw strong conclusions. The small sample size limits the generalizability of the findings and increases the risk of bias.

**Response:** We acknowledge the reviewer's concern regarding sample size and agree that larger cohorts enhance statistical power. However, our study employs rigorous statistical methods to ensure reliability, and our findings align with known biological mechanisms and prior research. While we recognize this as a limitation and have addressed it in a separate section, "Limitations of the study", our results provide valuable insights, and we are exploring opportunities for further validation in independent cohorts (Page number: 15;Line number :418-429).

**Comment 2:** The study presents a snapshot comparison between CFC and DT samples, but it does not track microbiome changes over time in chemotherapy patients. This limits the ability to assess whether changes in microbiota composition are transient or persist throughout treatment.

**Response:** Thank you for your insightful comment. Our study provides a comparative analysis between CFC and DT samples, offering a valuable snapshot of microbiome differences in chemotherapy patients. However, we acknowledge that the lack of longitudinal data limits our ability to determine whether these microbiota changes are transient or persist throughout treatment. Future studies incorporating time-course sampling would be essential to address this limitation and provide a more comprehensive understanding of microbiome dynamics during chemotherapy. We appreciate your suggestion and will include this point in the limitations of the study to highlight the need for longitudinal investigations (Page number: 15;Line number :418-429).

**Comment 3:** The study establishes microbiome differences between groups but does not thoroughly examine their clinical relevance, such as patient responses to chemotherapy or specific side effects.

**Response:** Thank you for your insightful comment regarding the clinical relevance of microbiome differences, particularly in relation to chemotherapy response and side effects. We acknowledge the importance of linking microbiome variations to patient outcomes, as it could provide valuable translational insights. In our current study, primary focus was to establish and characterize microbiome differences between groups, laying the groundwork for future investigations into their functional and clinical implications. While we recognize that direct correlations with chemotherapy response or specific side effects would strengthen the study's clinical impact, our dataset currently lacks detailed longitudinal clinical data to assess these associations robustly. However, the metadata includes information on cognitive impairment and depression as side effects.

However, we have expanded our discussion to highlight potential mechanistic links between the identified microbial signatures and known pathways involved in chemotherapy metabolism, immune modulation, and inflammation. We appreciate the reviewer's valuable suggestion, and we believe these additions enhance the contextual relevance of our findings while maintaining the study's primary scope.

The detailed metadata information was provided in the Supplementary information. The link is mentioned here

(<https://github.com/Initamizh/Microbiome_treatment_BC.git>).

**Comment 4:** The study identifies associations between microbiome composition and chemotherapy resistance (e.g., SOX8-Ruminococcus link) but does not experimentally validate these findings.

**Response:** Our study aimed to identify potential associations between microbiome composition and chemotherapy resistance, such as the *SOX8-Ruminococcus* link, using rigorous bioinformatics and statistical analyses. However, we acknowledge that experimental validation was not conducted within the scope of this study. Future work will focus on functional validation through *in vitro* and *in vivo* models to establish causality and mechanistic insights. We have revised the manuscript to clarify this limitation and highlight the need for further experimental validation (Page number: 14;Line number:385-400).

**Comment 5:** The study does not account for potential confounding factors such as diet, medication use or lifestyle differences, which could significantly influence microbiome composition.

**Response:** We appreciate the reviewer's concern regarding potential confounding factors such as diet, medication use, and lifestyle differences that could influence microbiome composition. However, we would like to clarify that this information was included in the original research paper in Table 1 and Table 2. These tables outline key metadata, including relevant confounders, to provide a comprehensive context for our analysis. To ensure clarity, we have now explicitly referenced these tables in the manuscript text and discussion section. We hope this revision addresses the concern and appreciate the opportunity to refine our work.

**Research paper:**

1.Bilenduke E, Sterrett JD, Ranby KW, Borges VF, Grigsby J, Carr AL, et al. Impacts of breast cancer and chemotherapy on gut microbiome, cognitive functioning, and mood relative to healthy controls. Scientific Reports. 2022 Nov 15;12(1).

**Comment 6:**The study identifies enriched pathways but does not extensively discuss their implications for chemotherapy metabolism or patient health.

**Response:** We appreciate the reviewer's insightful comment regarding the need for a more extensive discussion on the implications of enriched pathways in chemotherapy metabolism and patient health. While our primary focus was on identifying key pathways associated with chemotherapy resistance, we recognize the importance of contextualizing these findings in a broader biological and clinical framework.

To address this, we have substantially expanded our discussion to provide a more in-depth interpretation of how these pathways may influence drug metabolism, detoxification mechanisms, immune modulation, and tumor microenvironment interactions. For instance, the *Ruminococcus* genus has been linked to altered chemotherapy responses through modulation of host metabolism and immune interactions. *SOX8*, a transcription factor, is implicated in chemotherapy resistance, and its association with *Ruminococcus* could suggest a microbiome-driven resistance mechanism. We have also integrated additional references to strengthen the link between our findings and established mechanisms of chemotherapy resistance.

Furthermore, we acknowledge that our bioinformatics-driven approach provides valuable insights. We have revised the manuscript to emphasize these aspects and ensure a more comprehensive discussion of the clinical relevance of our findings (Page number : 13-14 ;Line number:379-403).

**Reviewer #2: Major concerns:**

**Comment 1:**The authors did a metagenomic analysis, it is recommended that the authors compare with other types of data like (16s) and strain specific date.

**Response:** We appreciate the reviewer's suggestion regarding strain-specific data and alternative data types. However, our study was specifically designed to assess gut microbiome diversity, community composition, and pathway analysis using 16S rRNA amplicon sequencing, a well-established and widely accepted approach for taxonomic classification at the genus and species levels.

While strain-level resolution requires whole-genome metagenomic sequencing, which was beyond the scope of this study, 16S rRNA amplicon sequencing remains a robust method for capturing microbial community shifts, particularly in response to chemotherapy. Our analysis effectively characterizes the microbiome differences between cancer-free controls (CFC) and women undergoing chemotherapy (DT), aligning with our study objectives.

Future investigations incorporating shotgun metagenomics or strain-specific approaches could further refine these findings, but our current methodology provides valuable insights into microbiome dynamics in this context.

**Reference paper:**

1.Bilenduke E, Sterrett JD, Ranby KW, Borges VF, Grigsby J, Carr AL, et al. Impacts of breast cancer and chemotherapy on gut microbiome, cognitive functioning, and mood relative to healthy controls. Scientific Reports. 2022 Nov 15;12(1).

**Comment 2:**It is recommended that the authors also include the normalization method for the data.

**Response:** We appreciate the reviewer's suggestion regarding the inclusion of the normalization method. In our study, we have used Total Sum Scaling (TSS) normalization, where each feature count is divided by the total sum of counts per sample to account for differences in sequencing depth. This method allows for direct comparison of relative abundances across samples. To enhance clarity, we have now explicitly mentioned this in the manuscript (Page number : 06; Line number :164-167).

**Comment 3:** The authors are recommended to acknowledge the limitations of small sample size in beta diversity analysis and adjust interpretations accordingly.

**Response:** We acknowledge the reviewer's concern regarding the limitations of a small sample size in beta diversity analysis. Our study includes 33 samples,we recognize that smaller sample sizes can impact the statistical power and generalizability of beta diversity metrics. To address this, we have taken the following steps:

**Statistical Considerations:** We applied appropriate distance metrics and statistical tests to ensure robust beta diversity comparisons, minimizing potential biases.

**Interpretation Adjustments:** We have carefully interpreted our beta diversity findings, emphasizing trends rather than definitive conclusions.

We have now explicitly acknowledged this limitation in the discussion and have updated our results accordingly.

**Comment 4:**The authors are recommended to explain the mechanistic link between gut microbiota and specific gene regulation in cancer treatment.

**Response:** *Ruminococcus* is a genus of bacteria commonly found in humans' gut microbiota. It plays a role in breaking down complex carbohydrates and producing short-chain fatty acids (SCFAs), which are important for gut health and overall metabolism. However, the direct association between *Ruminococcus* and the *SOX8* gene is not well-documented in scientific literature.

The *SOX8* gene is part of the SOX (SRY-related HMG-box) family of transcription factors, which are involved in various developmental processes, including sex determination, neurogenesis, and other regulatory functions. *SOX8*, in particular, has been studied in the context of brain development, spermatogenesis, and certain cancers.

The potential connection between *Ruminococcus* and *SOX8*, it might involve indirect mechanisms such as:

**1. Gut-Brain Axis:** The gut microbiota, including *Ruminococcus*, can influence brain function and gene expression through the gut-brain axis. Changes in gut microbiota composition could potentially affect the expression of genes like *SOX8* in the brain or other tissues.

**2. Metabolic Interactions:** *Ruminococcus* produces SCFAs like butyrate, which can influence host gene expression and epigenetic regulation. It is possible that these metabolites could indirectly affect *SOX8* expression.

**3. Immune System Modulation:** Gut bacteria like *Ruminococcus* can modulate the immune system, which in turn might influence gene expression in various tissues, including those where *SOX8* is active.

**Reference papers:**

1. Marchesi JR, Adams DH, Fava F, Hermes GDA, Hirschfield GM, Hold G, et al. The gut microbiota and host health: a new clinical frontier. Gut. 2015 Sep 2;65(2):330–9. Available from:

https://www.ncbi.nlm.nih.gov/pmc/articles/PMC4752653/

2.Mirzaei R, Dehkhodaie E, Bouzari B, Rahimi M, Gholestani A, Hosseini-Fard SR, et al. Dual role of microbiota-derived short-chain fatty acids on host and pathogen. Biomedicine & Pharmacotherapy. 2022 Jan;145:112352.

3.Xie S, Fan S, Zhang SY, Chen WX, Li QX, Pan G, et al. SOX8 regulates cancer stem-like properties and cisplatin-induced EMT in tongue squamous cell carcinoma by acting on the Wnt/β-catenin pathway. International Journal of Cancer. 2017 Nov 6;142(6):1252–65.

4.Carabotti M, Scirocco A, Maselli MA, Severi C. The gut-brain axis: interactions between enteric microbiota, central and enteric nervous systems. Annals of Gastroenterology. 2015 Apr 1;28(2):203–9. Available from: https://pubmed.ncbi.nlm.nih.gov/25830558/

5.Dalmasso G, Nguyen HTT, Yan Y, Laroui H, Charania MA, Ayyadurai S, et al. Microbiota Modulate Host Gene Expression via MicroRNAs. DeLeo FR, editor. PLoS ONE. 2011 Apr 29;6(4):e19293.

6.Martin AM, Sun EW, Rogers GB, Keating DJ. The Influence of the Gut Microbiome on Host Metabolism Through the Regulation of Gut Hormone Release. Frontiers in Physiology. 2019 Apr 16;10. Available from: https://www.ncbi.nlm.nih.gov/pmc/articles/PMC6477058/

7.Boccuto L, Tack J, Ianiro G, Abenavoli L, Scarpellini E. Human Genes Involved in the Interaction between Host and Gut Microbiome: Regulation and Pathogenic Mechanisms. Genes. 2023 Mar 31;14(4):857.

**Comment 5:** Authors are highly recommended to submit the custom scripts for data analysis on public databases(Github/zenodo).

**Response:** We appreciate the reviewer's recommendation regarding the submission of our custom scripts to public repositories for transparency and reproducibility. We have now made our scripts available on <https://github.com/Initamizh/Microbiome_treatment_BC.git>,ensuring accessibility for the research community. We have analyzed the data using MicrobiomeAnalyst tool. The respective input files were provided in the GitHub link. Additionally, we have included the repository link in the manuscript for reference.

**Comment 6:**Authors need to add more relevant citations for the mechanism.

**Response:** We appreciate the reviewer's suggestion to include more relevant citations for the discussed mechanism. We have now incorporated additional references that provide further support and context for our findings. These citations include key studies that elaborate on the molecular pathways and mechanisms involved. The revised manuscript now offers a more comprehensive and well-supported discussion (Page number : 14; Line number :385-400).

**Reference papers:**

1. Marchesi JR, Adams DH, Fava F, Hermes GDA, Hirschfield GM, Hold G, et al. The gut microbiota and host health: a new clinical frontier. Gut. 2015;65: 330–339. doi:https://doi.org/10.1136/gutjnl-2015-309990
2. Mirzaei R, Dehkhodaie E, Bouzari B, Rahimi M, Gholestani A, Hosseini-Fard SR, et al. Dual role of microbiota-derived short-chain fatty acids on host and pathogen. Biomedicine & Pharmacotherapy. 2022;145: 112352. doi:https://doi.org/10.1016/j.biopha.2021.112352
3. Xie S, Fan S, Zhang S-Y, Chen W-X, Li Q-X, Pan G, et al. SOX8 regulates cancer stem-like properties and cisplatin-induced EMT in tongue squamous cell carcinoma by acting on the Wnt/β-catenin pathway. International Journal of Cancer. 2017;142: 1252–1265. doi:https://doi.org/10.1002/ijc.31134
4. Dalmasso G, Nguyen HTT, Yan Y, Laroui H, Charania MA, Ayyadurai S, et al. Microbiota Modulate Host Gene Expression via MicroRNAs. DeLeo FR, editor. PLoS ONE. 2011;6: e19293. doi:https://doi.org/10.1371/journal.pone.0019293
5. Martin AM, Sun EW, Rogers GB, Keating DJ. The Influence of the Gut Microbiome on Host Metabolism Through the Regulation of Gut Hormone Release. Frontiers in Physiology. 2019;10. doi:https://doi.org/10.3389/fphys.2019.00428
6. Boccuto L, Tack J, Ianiro G, Abenavoli L, Scarpellini E. Human Genes Involved in the Interaction between Host and Gut Microbiome: Regulation and Pathogenic Mechanisms. Genes. 2023;14: 857. doi:https://doi.org/10.3390/genes14040857

**Minor concerns**

**Comment 1:** Authors need to attach clear figures(all).

**Response:** We have carefully reviewed all the figures and ensured that they are now presented with improved clarity.

**Comment 2:** The authors are recommended to use standardized numerical values.

**Response:** In response, we have carefully reviewed all numerical data in the manuscript and ensured consistency in the formatting and unit representation.

**Comment 3:** Authors need to remove the redundant phrases

**Response:** We have carefully revised the manuscript to eliminate unnecessary repetitions and improve conciseness while maintaining clarity and coherence.
